# Supplementary material for: KAN-GLNet: An enhanced PointNet++ model for canola silique segmentation and counting
Source: PLoS One. 2025 Nov 17;20(11):e0336622. doi: 10.1371/journal.pone.0336622 (PMC12622843; doi:10.1371/journal.pone.0336622)
Supplement: Attachment — Submitted filename: Response to Reviewers.docx [file pone.0336622.s001.docx]

**PLOS ONE**

**Manuscript ID：PONE-D-25-17533**

KAN-GLNet: An Enhanced PointNet++ Model for Canola Silique Segmentation and Counting

Jiajun Liu1 , Bei Zhou^1,2*^, Jie Liu^3^ , Xike Zhang^1^ , Jiangshu Wei^1,2^, Yao Zhang^1^ , Junjie

Wu^1^ , Changping Wu^3^ , Di Hu^1^

1 College of Information Engineering, Sichuan Agricultural University, Yaan 625000, China

2 Sichuan Key Laboratory of Agricultural Information Engineering, Sichuan Agricultural University, Yaan 625000, China

3 College of Agronomy, Sichuan Agricultural University, Chengdu 610000, China

* Corresponding author: [12801@sicau.edu.cn](mailto:12801@sicau.edu.cn)

**Based on the suggestions of several reviewers, we not only addressed each of the specific concerns they raised, but more importantly, we implemented a more rigorous division of the original dataset and conducted all experiments anew accordingly.**

① In our initial experiments, we expanded 50 original point clouds to 220 samples using data augmentation techniques. These were then split into training and testing sets for model training. The model demonstrated good fitting behavior during training and achieved high segmentation accuracy for canola siliques, with a mean Intersection over Union (mIoU) of 90.20%. In the subsequent clustering stage, the DBSCAN algorithm enabled us to achieve over 95% accuracy in silique counting. However, this experimental setup raised concerns from reviewers, mainly regarding the limited dataset size, the absence of a validation set (making it difficult to assess generalization and potential overfitting), and the risk of data leakage potentially inflating model performance.

② In response, we first divided the dataset into training, validation, and testing sets in a 7:2:1 ratio. We then applied data augmentation independently within each subset, expanding the dataset by a factor of 10, resulting in a total of 500 point clouds. Additionally, in the semantic segmentation stage, we introduced more recent state-of-the-art (SOTA) models such as SPG (2024) and PointMetabase (2023) for a comprehensive performance comparison. The results showed that during training, the loss curves for both training and validation sets exhibited good convergence, addressing concerns about the model’s generalization ability and overfitting. Furthermore, among all baseline models, ours achieved the highest segmentation accuracy (mIoU of 94.5%) with a relatively small number of parameters, fully demonstrating the superiority of our approach. Finally, in the clustering stage, we proposed a novel DBSCAN processing pipeline that more accurately identified individual siliques, achieving a silique recognition accuracy of 97.42%. These results underscore the advantages of our method and offer a promising new solution for agricultural scenarios, especially in resource-limited settings.

③In the revised manuscript, we will use the following formatting: reviewer comments in black italics, our responses in black regular font, and revisions in the manuscript in blue regular font, ensuring clear distinction.

**Response to Editor**

**Comment 1:** *Specifically, this work has only few images.*

**Authors Response:**

Dear Editor,

We sincerely appreciate your valuable feedback. Regarding the concern about limited image data, please allow us to provide a detailed clarification:

① Challenges in 3D Point Cloud Acquisition

Acquiring 3D point clouds is inherently more complex than capturing 2D images due to two key factors: the requirement for specialized sensors and data collection protocols, and the extensive manual annotation needed for intricate plant structures (Ma et al., Remote Sensing, 2019). For context, each rapeseed plant point cloud in our dataset was reconstructed from 120 images, meaning the 50-plant dataset effectively represents approximately 6,000 raw images.

② Limited Scale of Public Plant Point Cloud Datasets

Existing public datasets in this domain typically contain modest sample sizes. For example: ROSE-X consists of only 11 annotated rose bush models; Soybean-MVS includes 102 point clouds covering 5 soybean varieties across 13 growth stages; Pheno4D contains 204 point clouds (84 maize, 140 tomato); and PLANesT-3D has merely 34 point clouds (10 pepper, 10 rose, 14 ribes).

③ Proven Feasibility of Small-Scale Training

Prior research has successfully demonstrated plant phenotyping with limited data. Guo et al. (2023) achieved accurate leaf counting using only 60 augmented cabbage point clouds combined with an improved DBSCAN algorithm. Similarly, Ma et al. obtained good model convergence with just 50 eggplant seedling point clouds, confirming that meaningful analysis is possible even with constrained datasets.

④ Substantial Dataset Expansion in Revision

In response to your comments, we have significantly expanded our dataset to 500 point clouds (a 10-fold increase from the original), while rigorously preventing data leakage. Our models demonstrate excellent training/validation loss convergence and outperform all state-of-the-art benchmarks in accuracy metrics.

**Comment 2：***Large number of parameters, which is not proper for deep learning algorithm.*

**Authors Response:** We fully understand you are concern on this issue and sincerely appreciate this valuable comment. Below is our detailed explanation regarding this matter:

① Model Innovations

The core contribution of our work is the KAN-GLNet model, which introduces three key innovations: an inverted KAN convolution for enhanced feature extraction, a GLFN block for effective multiscale local-global information fusion, and a ContralNormal module that improves feature stability through contrastive learning. The model achieves superior performance (94.50% mIoU, 96.72% mAcc, 97.77% OAcc) compared to all baseline methods.

② Domain-Specific Methodological Requirements

In plant phenotyping research, deep learning segmentation alone is often insufficient. As established by Ferrara et al. (2018), downstream clustering algorithms like DBSCAN remain essential for organ-level analysis. Our work addresses this by: developing an optimized DBSCAN workflow with systematic parameter selection guidelines, and providing thorough evaluation of its application in silique clustering tasks.

We firmly believe this work not only meets deep learning research standards but also makes meaningful contributions to 3D plant phenotyping. We are deeply grateful for the editorial team's expertise and constructive suggestions, which have significantly improved our manuscript.

**Comment 3:** *Please ensure that your manuscript meets PLOS ONE's style requirements, including those for file naming.*

**Authors Response:** Thank you for your attention to this matter. Our manuscript was prepared using LaTeX and submitted in PDF format. The LaTeX template we used was downloaded from the PLOS ONE official website at the following link: https://journals.plos.org/plosone/s/latex#loc-references. Additionally, we have carefully checked the formatting of figures, tables, references, and other elements to ensure compliance with the requirements. If you have any questions or identify any issues with our formatting, we sincerely appreciate your feedback so that we can make further detailed revisions.

**Comment 4:** *Thank you for stating the following financial disclosure:*

*[This project is supported by National Natural Science Foundation of China, grant number 32301762.].Please state what role the funders took in the study.  If the funders had no role, please state: "The funders had no role in study design, data collection and analysis, decision to publish, or preparation of the manuscript."If this statement is not correct you must amend it as needed.*

*Please include this amended Role of Funder statement in your cover letter; we will change the online submission form on your behalf.*

**Authors Response:** Thank you for your attention to this work. We confirm that this project was supported by the National Natural Science Foundation of China, Grant No. 32301762, and that the funder participated in data collection and provided financial support.

**Comment 5:** *Please include a separate caption for each figure in your manuscript.*

**Authors Response:** We sincerely apologize for our oversight in the previous submission, where several figures were missing their corresponding titles. In our subsequent revisions, we have meticulously verified and ensured that every figure and table now has an appropriate title. Thank you very much for your understanding.

**Response to Reviewer 1:**

**Comment 1:** *The full forms of KAN-GLNet, DBSCAN, antd GLFN should be included at least once in the abstract, preferably at the beginning.*

**Authors Response:** Thank you for your valuable feedback. We sincerely appreciate bringing this oversight to our attention. The revised Abstract is now provided below in full, with all necessary corrections implemented.

**-----------------------------------------------------------------------------------------------------------------**

Accurate analysis of plant phenotypic traits is crucial for crop breeding and precision

agriculture. This study proposes a lightweight semantic segmentation model named

KAN-GLNet(Kolmogorov–Arnold Network with Global–Local Feature Modulation),

based on an enhanced PointNet++ architecture and integrated with an optimized

Density-Based Spatial Clustering of Applications with Noise (DBSCAN) algorithm, to

achieve high-precision segmentation and automatic counting of canola siliques. A

multi-view point cloud acquisition platform was built, and high-fidelity canola point

clouds were reconstructed using Neural Radiance Fields (NeRF) technology. The

proposed model includes three key modules: Reverse Bottleneck Kolmogorov–Arnold

Network Convolution, a Global–Local Feature Modulation (GLFN) block, and a

contrastive learning-based normalization module called ContraNorm. KAN-GLNet

contains only 5.72M parameters and achieves 94.50% mIoU, 96.72% mAcc, and 97.77%

OAcc in semantic segmentation tasks, outperforming all baseline models. In addition,

the DBSCAN workflow was optimized, achieving a counting accuracy of 97.45% in the

instance segmentation task. This method achieves an excellent balance between

segmentation accuracy and model complexity, providing an efficient solution for

high-throughput plant phenotyping. The code and dataset have been made publicly

available at: <https://anonymous.4open.science/r/KAN-GLNet-6432/.>

**-----------------------------------------------------------------------------------------------------------------**

**Comment 2:** *The introduction needs to be broadened by citing similar previous studies involving the algorithm in agriculture; additionally, the objectives should be framed based on the limitations of these related studies.*

**Authors Response:** We have carefully reviewed our previous manuscript and realized that the Introduction focused primarily on comparing our model's advantages while overlooking discussions of similar agricultural algorithms in prior studies. Accordingly, we have significantly expanded the Introduction to cover current research in this field, highlighting existing limitations that motivate our work. Specifically: Current plant phenotyping segmentation models struggle to balance performance and parameter efficiency, and we propose a novel point cloud segmentation model to address this trade-off. These revisions appear in Lines 40–73 of the revised manuscript.

**-----------------------------------------------------------------------------------------------------------------**

To address these challenges, deep learning-based point cloud segmentation methods have emerged, automatically learning features through data-driven approaches. Early research primarily adopted voxelization to convert point clouds into structured data for feature extraction ^[13]^. For example, Das Choudhury et al. ^[14]^ segmented maize stems and leaves using multi-view visual hull algorithms with voxel overlap verification and Euclidean clustering. Saeed et al. ^[15]^ implemented 3D segmentation of cotton stems, branches, and bolls via Point-Voxel Convolutional Neural Networks (PVCNN). However, these methods demand substantial computational resources and may incur information loss during segmentation. To overcome point cloud processing bottlenecks, subsequent research focused on direct point cloud processing architectures, mainly developing along two directions: point-based methods and Transformer-based approaches. The pioneering PointNet series ^[16, 17]^ laid the groundwork for point cloud deep learning, providing crucial technical support for plant 3D phenotyping. For instance, Ao et al. ^[18]^ applied PointNet for maize stem-leaf separation using local point density. Guo et al. ^[19]^ integrated PointNet++ with ASAP attention modules, achieving 86% mIoU in cabbage segmentation. Addressing PointNet++ optimization needs, PointNeXt achieved performance breakthroughs through training strategy updates and model scaling, with Dong et al. ^[20]^ reporting 89.21% mIoU (sugarcane), 89.19% mIoU (maize), and 83.05% mIoU (tomato) stem-leaf segmentation at 6.08M parameters. While such point-based models excel in parameter efficiency, they often face accuracy limitations due to insufficient feature extraction. PCT ^[21]^ pioneered Transformer architecture for 3D point cloud processing, marking its first application in point cloud segmentation. For example, Ma et al. ^[22]^ proposed PSTNet with cascaded self-attention (PSA) and local feature aggregation (NPA), achieving 92.20% IoU for eggplant point clouds. Yang et al. ^[23]^ developed PACANet, a Transformer-based pairwise attention center axis aggregation network, achieving 92.46% mean accuracy for maize populations at 46.2M parameters. These studies demonstrate that while Transformer architectures deliver breakthrough performance, they often suffer from parameter explosion, severely limitingdeployment in resource-constrained scenarios. To address the contradiction between parameter quantity and segmentation accuracy in plant point cloud segmentation, this study proposes KAN-GLNet, a

Kolmogorov-Arnold segmentation network equipped with global-local feature modulation. The model is designed to achieve high-precision segmentation under low-parameter constraints.

**Comment 3:** *Experimental results should not be included within the objectives of the study.*

**Authors Response:**Thank you for your correction. We have completed the replacement in the introduction section, specifically from lines 73 to 83, as shown below.

**-----------------------------------------------------------------------------------------------------------------**

The key innovations include:

(i)Constructing the first NeRF-derived rapeseed silique point cloud dataset (50

samples), expanding sample scale through data augmentation strategies.

(ii)Proposing reverse bottleneck KAN convolution (enhanced Kolmogorov-Arnold

convolution) to replace conventional convolutions for strengthening feature extraction

capability.

(iii)Designing GLFN multi-scale feature modulation blocks to optimize spatial

representation of complex silique structures by fusing global-local attention mechanisms.

(iv)Introducing the ContraNorm contrastive normalization module to enhance point

cloud feature stability and segmentation consistency.

(v)Proposing an optimized DBSCAN workflow algorithm to achieve automated

silique instance counting.

**-----------------------------------------------------------------------------------------------------------------**

**Comment 4:** *The source of image acquisition must be clearly stated, as it is critical to dataset quality. Specifications should be tabulated for better readability. Further, both natural and artificial environmental conditions used for dataset creation must be elaborated. The following reference should be cited for improving dataset acquisition presentation. Paul, A., Machavaram, R., Kumar, D., & Nagar, H. (2024). Smart solutions for capsicum Harvesting: Unleashing the power of YOLO for Detection, Segmentation, growth stage Classification, Counting, and real-time mobile identification. Computers and Electronics in Agriculture, 219, 108832.*

**Authors Response:**Thank you for bringing this issue to our attention. Our rapeseed was cultivated in the experimental fields of Sichuan Agricultural University in Ya'an, China. The field pods were extremely dense, with severe overlapping. We transplanted the rapeseed from the field into pots for subsequent phenotypic extraction on our designed indoor rapeseed phenotyping platform.

Following your suggestion, we have addressed the natural environment for dataset creation by including images of the rapeseed growth conditions in the experimental fields in the "Limitations" section of the discussion. Additionally, for the more critical description of imaging parameters, we have referenced the literature you provided to meticulously present the artificial environment during image capture.The detailed description can be found in the Data Collection section, lines 101–115.

**-----------------------------------------------------------------------------------------------------------------**

Data collection employed an Apple iPhone 15 Pro Max smartphone featuring a 48-megapixel main camera with an f/1.78 aperture and 24mm focal length. The study involved standardized acquisition of 50 canola plants, each positioned centrally on a turntable rotating at a constant angular velocity of 6 degrees per second. The camera maintained a fixed horizontal distance of 50 centimeters from the subject, systematically capturing video recordings from two distinct perspectives: a horizontal view and a 60-degree downward-tilted view. Each perspective was recorded for one minute, yielding two minutes of total footage per plant. The description of acquisition parameters was inspired by ^[25]^, particularly in terms of presenting camera specifications and shooting settings. Complete parameter details are provided in Table 1.

For image processing, FFmpeg software ^[26]^ extracted keyframes at one-second intervals, generating 120 frames per plant at a resolution of 3840 by 2160 pixels in JPG format, suitable for subsequent 3D reconstruction and analysis. The turntable’s continuous rotation protocol ensured optimal imaging stability while effectively preventing motion blur artifacts.

**-----------------------------------------------------------------------------------------------------------------**

Note:[25] Paul A, Machavaram R, Kumar D, Nagar H, et al. Smart solutions for capsicum

Harvesting: Unleashing the power of YOLO for Detection, Segmentation, growth stage Classification, Counting, and real-time mobile identification. Computers and Electronics in Agriculture. 2024;219:108832.

**Comment 5:** *NeRFStudio must be properly cited using an appropriate and complete citation format.*

**Authors Response:** Due to our oversight, we failed to notice that the abbreviation in this citation should be supplemented with its full name, which has now been added.

**Comment 6:** *The term "4.4-fold expansion" in dataset processing must be technically described, indicating the specific augmentation or data multiplication steps applied.*

**Authors Response:** We sincerely appreciate your guidance. During the revision stage, we redesigned the experiments, and a summary of the experimental workflow and results is provided at the beginning of this letter. The specific data augmentation methods, their sources of inspiration, and supporting research are described in detail in the Experiments section (Lines 324–343) to ensure the rigor of the study. To enhance the generalization ability of the model, we adopted a commonly used 7:2:1 ratio to divide the dataset into training, validation, and test sets. This division strategy is widely applied in related research.

**-----------------------------------------------------------------------------------------------------------------**

The difficulty of acquiring 3D point cloud data is significantly higher than that of 2D

images, which is mainly reflected in two aspects: first, it requires specialized sensors and

specific acquisition methods, and second, the complex structure of plants necessitates

extensive manual annotation efforts ^[42]^. Data augmentation techniques can effectively

alleviate the issue of insufficient training data, providing ample data support for model

optimization. The PointNext study confirmed the effectiveness of basic augmentation

strategies such as rotation and translation in improving model performance. Yao et

al. ^[43]^ employed random point deletion, noise addition, and scaling for point cloud data

of tomato plants across three key growth stages (seedling, flowering, and fruiting). Xie

et al. ^[44]^ successfully expanded the dataset size to 15 times its original scale by

applying operations such as point cloud cropping and jittering to public datasets like

Plant3D ^[45]^.

Based on previous research experience and computational efficiency considerations,

this study adopts a rigorous data processing workflow: first, the NeRF canola dataset is

divided into training, validation, and test sets at a ratio of 7:1:2, followed by 10-fold

data augmentation for each subset. Splitting the dataset before performing

augmentation is to mitigate potential data leakage risks. The specific augmentation

strategy includes four dimensions: randomly discarding 0–30% of point cloud data,

random rotation along the Z-axis between -180°and 180°, random scaling at a ratio of

0.9–1.1, and a 20% probability of flipping along the Y-axis.

**-----------------------------------------------------------------------------------------------------------------**

**Comment 7:** *Equation numbers must be cited properly in the text, similar to how figures and tables are referenced.*

**Authors Response:** We have revised all formula citations to reference them in the same manner as figure citations. The specific modifications are distributed throughout the Methodology section (Lines 178–281).

**Comment 8:** *The dataset division into only training and testing sets is inadequate. The absence of a validation set is a limitation. The rationale behind choosing 180 training points and 40 test points should be justified. Additionally, methods like cross-validation can be used to obtain more reliable performance estimates.*

**Authors Response:** Dear Reviewer, we sincerely appreciate your attention to this issue. In our previous study, we employed the classic S3DIS dataset for point cloud segmentation, which consists of 271 indoor scene point clouds from six areas (Area_1 to Area_6). The original scheme divided the data into training and test sets, using downsampled sub-point clouds from the test set as the validation set during the validation phase, with final evaluation performed on the complete test set.

As you pointed out, we recognized the limitations of this partitioning approach, particularly the lack of a dedicated validation set, which made it difficult to accurately assess model generalizability and overfitting risks. Accordingly, we have improved the data partitioning scheme: first dividing the data into training, validation, and test sets at a conventional ratio of 7:2:1, followed by 10-fold data augmentation within each subset (partitioning before augmentation to prevent data leakage).

As shown in Response 10, the loss and accuracy curves demonstrate good model fitting, effectively alleviating concerns about generalization capability. Given the current rigorous data partitioning, sufficient data augmentation, and stable model performance, we have not yet implemented cross-validation on the raw point clouds. However, your valuable suggestions will be incorporated into future research to further refine the validation scheme and enhance the robustness of our study.

Once again, we sincerely thank you for your professional guidance!

**Comment 9:** *Software and hardware specifications used for training should be presented in a table to enhance clarity and readability.*

**Authors Response:** The figure is shown in Table 3.

**Comment 10(1):** *The choice of 300 training epochs must be justified based on the convergence behavior of the loss curves. Include loss curve plots to illustrate the training dynamics.*

**Authors Response:** Dear Reviewer,Based on the newly added validation set and the adjusted dataset partitioning scheme, we have systematically determined through experiments that 250 training epochs are optimal, as this setting ensures the model is fully trained to complete convergence. The relevant experimental evidence (including the convergence behavior of the loss curves) has been detailed in the "Semantic Segmentation Results" section (Lines 354–357) of the main text.

**-----------------------------------------------------------------------------------------------------------------**

KAN-GLNet exhibited a clear convergence trend in both training and validation losses.

As shown in Fig 8, the loss dropped rapidly during the first 100 epochs, indicating a fast

parameter optimization process. Subsequently, the loss curves gradually stabilized, with

the validation loss reaching a low plateau while the training loss continued to decline

slowly, ultimately achieving full convergence at epoch 250.

**-----------------------------------------------------------------------------------------------------------------**

Fig 8

**Comment 10(2):** *Also, provide the rationale for choosing a batch size of 8 and a learning rate of 0.01.*

**Authors Response:** During the revision phase, we modified the data partitioning and further enhanced the data augmentation. The previous hyperparameters were not suitable for our current task. Ultimately, we selected a learning rate of 0.0001 and a batch size of 16.

Since this study is essentially exploratory in nature, the selection of hyperparameters was mainly based on previous literature and empirical validation. Specifically, we referred to similar works, such as:

1.Yang, X., Miao, T., Tao, Y., Zhang, B., Wu, X., Han, X., ... & Xu, T. (2025). PACANet: A Paired-Attention Central Axis Aggregation Network for Plant Population Point Cloud Segmentation and Phenotypic Trait Extraction—A Case Study on Maize. Computers and Electronics in Agriculture, 237, 110611. The authors performed point cloud segmentation and subsequent phenotypic extraction on a self-constructed maize population dataset, where the initial learning rate of the segmentation model was set to 0.0001, though the batch size was not specified.

2.Han, B., Li, Y., Bie, Z., Peng, C., Huang, Y., & Xu, S. (2022). MIX-NET: Deep Learning-Based Point Cloud Processing Method for Segmentation and Occlusion Leaf Restoration of Seedlings. Plants, 11(23), 3342. The authors' experiments on a public point cloud dataset showed that the combination of batch size = 16 and LR = 0.0001 effectively balanced segmentation accuracy and computational efficiency. This finding aligns well with the hardware conditions of our study.

3.Cheng, X., Lei, R., Huang, D., Liao, Z., Piao, F., Chen, Y., Feng, P., & Zeng, L. (2024). Constraint-Aware Feature Learning for Parametric Point Cloud. arXiv preprint arXiv:2411.07747v4. https://arxiv.org/pdf/2411.07747v4. The authors adopted exactly the same parameter settings (batch size = 16, LR = 0.0001) on the Param20K dataset, and the resulting SOTA performance further supports the applicability of this parameter combination in the point cloud domain.

These studies used similar configurations in related tasks. Considering the trade-off between model accuracy and computational efficiency, and based on these references as well as several trial runs within a reasonable range, we found that the current parameter settings yield stable and promising results. Although this version of our study has not yet conducted comprehensive ablation experiments or sensitivity analysis, we believe the reported experimental settings are representative and the results obtained under the stated conditions are reproducible. We appreciate your suggestion and will consider incorporating a more systematic evaluation scheme in future research.

**Comment 11:** *Results shown in Table 4 and Table 5 must be discussed in detail within the text, elaborating on the significance and implications of the values presented.*

**Authors Response:** Due to our modifications in the experimental design and the inclusion of additional statistical tables, the previous Table 4 and Table 5, which represented the ablation study and the effects of replacing different layers with inverted bottleneck KAN convolutions, respectively, now correspond to Table 6 and Table 8. The corresponding figures are shown below, and the detailed analyses can be found in the Ablation Experiment subsection and the Convolution Replacement Position subsection of the Experiments section.

**-----------------------------------------------------------------------------------------------------------------**

In this study, we systematically evaluated the individual and combined effects of three

key modules: Reverse Bottleneck KAN Convolutions, GLFN, and ContraNorm. These

modules were integrated into the baseline PointNet++ model both independently and

jointly for thorough validation. Table 6 presents a comparison of segmentation

performance across different module combinations on the NeRF canola point cloud

dataset.

The experimental results show that introducing the Reverse Bottleneck KAN

Convolutions leads to the most significant improvement in segmentation performance.

This demonstrates that the module is highly effective in extracting spatial structural

features, enabling the network to better recognize complex geometric boundaries such as

the junctions between siliques and stems, while maintaining a relatively low number of

parameters. The GLFN module enhances the model’s ability to perceive spatial details

by fusing multi-scale global and local contextual information. ContraNorm improves the

overall discriminative capability by introducing a contrastive learning mechanism that

alleviates feature degradation and reduces overlap between different classes.

It is worth noting that when all three modules are combined to form the complete

KAN-GLNet architecture, the model achieves the highest performance in terms of mean

Intersection over Union and overall accuracy, indicating improved segmentation

precision and stronger recognition of dominant structural components. However, the

mean class accuracy is slightly lower than that of the model without the GLFN module.

This is likely because the adaptive modulation in GLFN strengthens the fusion of global

and local features, but at the same time introduces feature bias, which reduces the

model’s attention to minority class samples and ultimately results in a slight drop in

mean class accuracy.

**-----------------------------------------------------------------------------------------------------------------**

**-----------------------------------------------------------------------------------------------------------------**

This paper introduces the Reverse Bottleneck KAN Convolution and applies it to the

Min-PointNet part of the PointNet++ model by replacing the first convolutional layer,

resulting in the design of KGL-PointNet. Table 8 presents the impact of replacing

convolution layers at shallow, deep, and multiple levels on model performance and

parameter count. Experimental results show that when the Reverse Bottleneck KAN

Convolution is placed at the shallowest layer of the feature extraction network, the

model achieves the highest mIoU, mAcc, and OAcc, with only a slight increase in

parameters compared to replacing the second layer. This indicates that shallow layers

are more effective at capturing low-level geometric features and local texture

information in the point cloud, while deeper layers focus more on semantic-level

representations, where the advantages of the Reverse Bottleneck KAN Convolution

become less pronounced.

Moreover, stacking multiple Reverse Bottleneck KAN Convolutions does not further

improve segmentation performance and instead leads to a decline. The primary reason

lies in the significant increase in parameter count, which introduces risks of overfitting

and increased computational burden. On the one hand, a large number of parameters

makes the network more prone to falling into local minima; on the other hand, excessive

feature enhancement may result in unstable gradient updates or redundant information,

thereby weakening the model’s generalization ability. Therefore, the optimal strategy is

not to simply increase the number of modules but to analyze the importance of features

at different layers in depth, adopting selective replacement or dynamic configuration

methods to maximize the effectiveness of shallow-layer geometric feature extraction

while maintaining model compactness.

**-----------------------------------------------------------------------------------------------------------------**

**Comment 12:** *Figure 10 is blurry and lacks clarity; it should be reworked for improved visibility and resolution.*

**Authors Response:** The previous Figure 10, which depicted the model training loss and Kyoto variation curves, now corresponds to the current Figure 8, showing the model training loss and Kyoto variation curves. The updated figure is presented below.

Fig 8

**Comment 13:** *The results of the current study should be critically compared with outcomes from similar previous studies to contextualize its contributions.*

**Authors Response:** This comparison has been provided in the Analysis of Experimental Results subsection of the Discussion section, with the detailed text located on pages 544–568.

**-----------------------------------------------------------------------------------------------------------------**

During the silique maturation stage, canola siliques are arranged in a disorderly and

heavily overlapping manner, while the number of branches varies significantly, posing

considerable challenges for segmentation. KAN-GLNet effectively addresses these

difficulties, successfully achieving precise segmentation, and the subsequently optimized

DBSCAN algorithm effectively resolves the issue of numerous silique outliers being

regarded as false clusters, providing a solid foundation for accurate counting and

phenotypic analysis.

In the field of plant point cloud semantic segmentation, commonly used models are

based either on point-based architectures or transformer architectures, both of which

struggle to achieve an excellent balance between model accuracy and parameter size.

For example, Dong et al. ^[20]^ proposed a segmentation model with a relatively low

parameter count of 6.08M, achieving stem and leaf segmentation accuracies of 89.21%

mIoU on sugarcane, 89.19% mIoU on maize, and 83.05% mIoU on tomato,

demonstrating the potential of lightweight design. However, the relatively lower

segmentation accuracy limits the precision of subsequent fine-grained phenotypic

extraction. On the other hand, Ma et al. ^[22]^ proposed PSTNet, a transformer-based

model that achieved 92.20% IoU on eggplant point clouds, but its large parameter size

results in high computational cost and deployment difficulty, which is unfavorable for

resource-constrained practical scenarios.

Our proposed model KAN-GLNet achieved 94.50% mIoU, 96.72% mAcc, and 97.77%

OAcc on the NeRF canola test set, significantly outperforming the second-place SPG

model based on PTV2 in terms of accuracy. Meanwhile, our model has only 5.72M

parameters, which is far fewer than most Transformer-based models, reflecting an

excellent balance between model accuracy and parameter size. This not only ensures

high segmentation accuracy but also greatly reduces the computational resource

requirements of the model, providing an efficient point cloud processing solution for

agricultural edge devices.

**-----------------------------------------------------------------------------------------------------------------**

**Comment 14:** *The limitations of the study should be clearly outlined in the second paragraph of the conclusion to offer a balanced perspective on the work.*

**Authors Response:** Dear Reviewer,We sincerely appreciate your observation regarding the lack of in-depth discussion on limitations in our initial draft. While we have carefully considered your suggestion, we ultimately chose to place the limitations analysis specifically in the Discussion section for a more thorough exploration. We believe this arrangement more effectively stimulates readers' critical reflection and provides clearer insights for future research. The detailed content can be found on pages 569–596 of the main text.

**-----------------------------------------------------------------------------------------------------------------**

In this study, we acquired point clouds of canola plants using a self-designed multi-view

image acquisition platform and applied KAN-GLNet for segmentation and clustering to

extract silique counts. However, this process still faces two major limitations that need

to be addressed.

As shown in Fig. 12, canola plants growing naturally in field plots are dense and

crowded, with severe silique overlap, making it difficult to capture complete multi-view

images without damaging the overall plant structure. Although we transplanted

individual plants into pots for imaging and used a low-speed turntable and uniform

lighting to ensure data quality to some extent, this process inevitably altered the

natural growth state of branches and leaves. As a result, the acquired point clouds differ

from those in real field conditions, affecting the accuracy of segmentation and clustering

outcomes in reflecting phenotypic traits under natural environments. Therefore, future

work may explore non-contact aerial or multi-angle scanning technologies such as drones

or mobile robotic arms to minimize plant structure interference. Alternatively, future

research could shift focus from individual canola plants to large-scale field-level

phenotypic estimation.

Our designed KAN-GLNet achieved 94.50% segmentation accuracy on the NeRF

canola dataset, a significant improvement over the baseline PointNet++, which achieved

only 70.40%, and it also outperformed other SOTA models. However, this improvement

came with a parameter increase from the baseline’s 1M to 5.7M, posing challenges for

deployment on resource-constrained edge devices or in real-time processing scenarios.

To balance high accuracy with real-time performance, further research is needed on

model compression and lightweight optimization techniques, such as weight

quantization, channel pruning, or knowledge distillation, to reduce model complexity

while maintaining segmentation performance, providing a viable pathway for future

applications in agricultural drone inspection, intelligent greenhouses, and mobile

terminals.

**-----------------------------------------------------------------------------------------------------------------**

Fig. 12

**Once again, we sincerely thank you for your thoughtful comments and constructive suggestions, which have been invaluable in improving the quality and clarity of our manuscript.**

**Response to Reviewer 2:**

**Comment :** *While the proposed method demonstrates promising segmentation and counting capabilities, the quantitative result visualizations in the current form lack clarity and are difficult to interpret. Moreover, the reliance on the DBSCAN clustering algorithm for instance separation raises concerns about its robustness across diverse plant samples, particularly in densely packed or overlapping regions. It is unclear whether DBSCAN consistently yields correct instance counts for all cases. To strengthen the work, the authors should provide clearer, high-resolution visualization of results and include statistical metrics—such as mean absolute error (MAE), root mean squared error (RMSE), and count accuracy—to quantitatively validate the reliability of the predicted instance counts across the dataset. This would provide a more rigorous and interpretable evaluation of their approach.*

**Authors Response:** Dear Reviewer,Thank you for your meticulous review of the key aspects of this study. In response to your valuable suggestions, we have made systematic improvements in the following three areas:

1.Algorithm Optimization: This section first demonstrates the general applicability of DBSCAN in plant point cloud processing, followed by an analysis of the specific clustering challenges presented by canola silique point clouds. Based on this, we restructured the application process of DBSCAN in plant point cloud clustering and proposed an improved DBSCAN framework incorporating SOR filtering (see pages 288–309, section “Canola Silique Recognition Based on the DBSCAN Algorithm”).

2.Visualization Enhancement: We added Fig. 10 (clustering effects under different hyperparameters) and Fig. 11 (comparison of the improved method), which present high-resolution visualizations of instance segmentation results. The detailed parameter optimization process is described on pages 485–519 in the section “DBSCAN Optimal Parameter Search and SOR Filter Necessity”.

3.Quantitative Validation: We fully introduced three evaluation metrics—Mean Absolute Error (MAE), Root Mean Square Error (RMSE), and Counting Accuracy (CA)—to statistically analyze the optimized method. The experimental results show that the optimized DBSCAN approach achieved a Mean Absolute Error (MAE) of 4.80, a Root Mean Square Error (RMSE) of 5.25, and a Counting Accuracy (CA) of 97.45% in the canola silique point cloud clustering stage. For details, please refer to the “Canola Silique Clustering Experiment” subsection on pages 394–416.

We are grateful for your constructive feedback, which has helped us enhance both the technical robustness and presentation clarity of this study. The updated experimental data, visualizations, and comprehensive analyses in the specified sections demonstrate how your suggestions have been implemented to strengthen our methodology and results. This revision process has not only addressed your specific concerns but also improved the overall quality of our research on canola phenotyping through advanced point cloud processing techniques.

Fig 10

Fig 11

Canola Silique Recognition Based on the DBSCAN Algorithm

**-----------------------------------------------------------------------------------------------------------------**

In plant phenotyping studies, applying clustering algorithms to the silique point clouds

extracted after semantic segmentation is a common approach for achieving organ-level

3D point cloud recognition ^[40]^. Elnashef et al. ^[41]^ employed the DBSCAN algorithm

and utilized local point cloud density features to accomplish high-precision instance

segmentation of the stem-leaf structures in dicotyledonous plants. Guo et al. ^[19]^, based

on semantic segmentation of seedling-stage cabbage point clouds, combined DBSCAN

with color filtering and edge filtering to successfully extract a variety of phenotypic

traits.

However, directly applying the above clustering methods to canola silique point

clouds still faces significant challenges. First, canola siliques often appear densely

clustered, interlaced, and heavily overlapped, making it difficult to distinguish between

adjacent clusters. Second, the semantic segmentation stage inevitably involves some

misclassification between siliques and stems. These incorrect labels introduce additional

noise into the clustering input, and outlier points further disrupt cluster connectivity.

Given that canola silique point clouds often contain outliers caused by segmentation

errors, occlusions, or background interference, directly performing clustering tends to

result in blurred or incorrect cluster boundaries. Therefore, to improve the stability and

robustness of the clustering stage, we introduced Statistical Outlier Removal (SOR)

filtering after semantic segmentation to clean the silique point cloud before applying

DBSCAN clustering. This process effectively improves the quality of the point cloud

prior to clustering and reduces the risk of outlier noise being misidentified as siliques.

**-----------------------------------------------------------------------------------------------------------------**

DBSCAN Optimal Parameter Search and SOR Filter Necessity

**-----------------------------------------------------------------------------------------------------------------**

To determine the optimal DBSCAN parameter combination suitable for the clustering

task of canola siliques, this study adopts a grid search method to systematically

evaluate the impact of two key parameters—neighborhood radius (eps) and minimum

number of samples (min samples)—on clustering performance. The parameter search

range was set as eps ∈ [0.05, 0.20] and min samples ∈ [1, 15]. As shown in Fig. 10, the

experimental results indicate that when the parameter combination is set to eps = 0.10

and min samples = 5, the optimized DBSCAN demonstrates the best clustering

performance in regions where the density of siliques varies significantly.

The study found that a smaller eps value effectively distinguishes neighboring silique

clusters that are spatially close, thereby avoiding under-segmentation. However, due to

uneven point cloud density, it may also lead to some siliques being misclassified as noise.

On the other hand, a moderate min samples value not only suppresses

over-segmentation caused by noise or residual points from semantic segmentation but

also avoids mistakenly discarding true silique points when the threshold is set too high.

Since raw silique point clouds often contain acquisition noise and segmentation

errors, directly applying the standard DBSCAN tends to misclassify these outliers as

independent clusters, resulting in the generation of false clusters and over-segmentation.

This negatively impacts the stability and repeatability of subsequent instance counting

and morphological analysis.As shown in Fig. 11, standard DBSCAN often counts

outliers as false clusters, whereas the improved method effectively addresses this issue.

By introducing statistical outlier removal (SOR) filtering into the clustering pipeline,

we first eliminate the most sparsely distributed points caused by segmentation residuals

and acquisition noise, thereby making silique cluster boundaries clearer and enhancing

their connectivity. Subsequently, using the optimized parameters of eps = 0.10 and

min samples = 5, clustering is performed on the remaining point cloud. This allows for

an adaptive balance between intra-cluster connectivity and inter-cluster separation in

both dense and sparse regions.

As a result, the generation of false small clusters is significantly reduced, and each

real silique cluster can be accurately extracted, greatly enhancing the robustness and

consistency of the clustering results. Ultimately, this improved pipeline proves

particularly effective in extracting phenotypic parameters from plants with complex and

overlapping structures such as fruits and leaves. It provides a reliable data foundation

for high-throughput quantification of canola siliques, reduces the burden of manual

correction, and offers solid algorithmic support for subsequent three-dimensional organ

morphological studies.

**-----------------------------------------------------------------------------------------------------------------**

Canola Silique Clustering Experiment

**-----------------------------------------------------------------------------------------------------------------**

The DBSCAN algorithm relies on two critical hyperparameters. The neighborhood

radius eps defines the maximum distance within which points are considered neighbors,

while the minimum number of samples min samples specifies the minimum number of

neighboring points required for a point to be classified as a core point. In this study, we

selected eps = 0.10 and min samples = 5 as the final parameter combination for silique

instance counting in canola plants.

We compared the clustering results with manual measurements to evaluate the

accuracy of the improved DBSCAN algorithm in instance-level silique counting. As

shown in Table 5 , the silique counts for individual canola plants exhibited strong

consistency. The overall evaluation metrics were as follows: a Mean Absolute Error

(MAE) of 4.80, a Root Mean Square Error (RMSE) of 5.25, and a Counting Accuracy

(CA) of 97.45%. These three metrics respectively validate the reliability of the clustering

results in terms of error magnitude, prediction stability, and overall accuracy, indicating

that the improved method can effectively reflect the actual number of plant organs.

Building on the original DBSCAN clustering process, we introduced SOR filtering to

remove local noise introduced during the semantic segmentation stage, thereby

improving the purity of the point cloud input. This enhancement significantly increases

the robustness of DBSCAN in densely entangled regions, enabling it to accurately

separate neighboring organs even under severe clustering, occlusion, and overlap of

siliques. The results demonstrate that our proposed clustering pipeline is not only

simple and efficient but also well-suited to the high-precision and high-adaptability

demands of organ-level recognition in plant phenotyping.

**-----------------------------------------------------------------------------------------------------------------**

**Once again, we sincerely thank you for your thoughtful comments and constructive suggestions, which have been invaluable in improving the quality and clarity of our manuscript.**

**Response to Reviewer 3:**

**Comment 1:** *The manuscript introduces a modified model architecture for pointcloud segmentation tasks. While the proposed model is well described and the authors provide detailed explanations, several important concerns limit the strength of the conclusions and the reliability of the results. Dataset Size vs. Model Complexity: The model contains over 5 million parameters but is trained on a dataset consisting of only 50 original samples, augmented to a total of 220. This is a very small dataset for such a large model, and the manuscript does not provide evidence that steps were taken to mitigate overfitting.*

**Authors Response:** Dear Reviewer, thank you for your attention to this part of our work. As we explained to the editor regarding the limited number of images, plant point cloud data is generally difficult to obtain, and even a small number of samples can be used for model training.please allow us to provide a detailed clarification:

1.Challenges in 3D Point Cloud Acquisition

Acquiring 3D point clouds is inherently more complex than capturing 2D images due to two key factors: the requirement for specialized sensors and data collection protocols, and the extensive manual annotation needed for intricate plant structures (Ma et al., Remote Sensing, 2019). For context, each rapeseed plant point cloud in our dataset was reconstructed from 120 images, meaning the 50-plant dataset effectively represents approximately 6,000 raw images.

2.Limited Scale of Public Plant Point Cloud Datasets

Existing public datasets in this domain typically contain modest sample sizes. For example: ROSE-X consists of only 11 annotated rose bush models; Soybean-MVS includes 102 point clouds covering 5 soybean varieties across 13 growth stages; Pheno4D contains 204 point clouds (84 maize, 140 tomato); and PLANesT-3D has merely 34 point clouds (10 pepper, 10 rose, 14 ribes).

3.Proven Feasibility of Small-Scale Training

Prior research has successfully demonstrated plant phenotyping with limited data. Guo et al. (2023) achieved accurate leaf counting using only 60 augmented cabbage point clouds combined with an improved DBSCAN algorithm. Similarly, Ma et al. obtained good model convergence with just 50 eggplant seedling point clouds, confirming that meaningful analysis is possible even with constrained datasets.

4.Substantial Dataset Expansion in Revision

In response to your comments, we have significantly expanded our dataset to 500 point clouds (a 10-fold increase from the original), while rigorously preventing data leakage.

Furthermore, regarding our model size exceeding 5 million parameters (actually 5.72M), we have systematically reviewed related studies in the Introduction section, pages 48–84. In summary, plant point cloud segmentation methods based on points can be broadly categorized into point-based models and Transformer-based models. The former generally have fewer parameters but face limitations in segmentation accuracy. For example, Dong et al. ^[20]^ achieved mIoUs of 89.21%, 89.19%, and 83.05% for sugarcane, maize, and tomato stem-leaf segmentation tasks, respectively, with a model size of 6.08M parameters. In contrast, Transformer-based methods offer higher segmentation accuracy but often suffer from a bottleneck in balancing parameter size and accuracy. For instance, Yang et al. ^[23]^ developed the Transformer-based PACANet, which utilizes pairwise attention and center-axis feature aggregation, achieving a mean accuracy of 92.46% in maize population segmentation, but with a model size as large as 46.2M parameters. To address this trade-off between parameter quantity and accuracy, we propose KAN-GLNet, a Kolmogorov-Arnold segmentation network integrating global-local feature modulation, designed to achieve high-precision point cloud semantic segmentation under low-parameter constraints. Subsequent experimental results clearly demonstrate our model’s excellent segmentation performance under the dual constraints of accuracy and parameter size.For detailed descriptions, please refer to pages 40–84 in the Introduction section.

Finally, as shown in Fig. 8, KAN-GLNet’s training and validation losses clearly converged, with a rapid decline in the first 100 epochs indicating fast parameter optimization. Afterwards, the losses stabilized, with validation loss remaining low and training loss decreasing slowly, reaching full convergence at epoch 250. The gap between training and validation losses and accuracy curves was minimal, mainly due to the limited number of original samples (only 45). Overall, KAN-GLNet demonstrated strong accuracy and effectively mitigated overfitting in canola organ segmentation despite the limited sample size.

**-----------------------------------------------------------------------------------------------------------------**

To address these challenges, deep learning-based point cloud segmentation methods

have emerged, automatically learning features through data-driven approaches. Early

research primarily adopted voxelization to convert point clouds into structured data for

feature extraction ^[13]^. For example, Das Choudhury et al. ^[14]^ segmented maize stems

and leaves using multi-view visual hull algorithms with voxel overlap verification and

Euclidean clustering. Saeed et al. ^[15]^ implemented 3D segmentation of cotton stems,

branches, and bolls via Point-Voxel Convolutional Neural Networks (PVCNN). However,

these methods demand substantial computational resources and may incur information

loss during segmentation. To overcome point cloud processing bottlenecks, subsequent

research focused on direct point cloud processing architectures, mainly developing along

two directions: point-based methods and Transformer-based approaches. The pioneering

PointNet series ^[16, 17]^ laid the groundwork for point cloud deep learning, providing

crucial technical support for plant 3D phenotyping. For instance, Ao et al. ^[18]^ applied

PointNet for maize stem-leaf separation using local point density. Guo et al. ^[19]^

integrated PointNet++ with ASAP attention modules, achieving 86% mIoU in cabbage

segmentation. Addressing PointNet++ optimization needs, PointNeXt achieved

performance breakthroughs through training strategy updates and model scaling, with

Dong et al. ^[20]^ reporting 89.21% mIoU (sugarcane), 89.19% mIoU (maize), and 83.05%

mIoU (tomato) stem-leaf segmentation at 6.08M parameters. While such point-based

models excel in parameter efficiency, they often face accuracy limitations due to

insufficient feature extraction. PCT ^[21]^ pioneered Transformer architecture for 3D

point cloud processing, marking its first application in point cloud segmentation. For

example, Ma et al. ^[22]^ proposed PSTNet with cascaded self-attention (PSA) and local

feature aggregation (NPA), achieving 92.20% IoU for eggplant point clouds. Yang et

al. ^[23]^ developed PACANet, a Transformer-based pairwise attention center axis

aggregation network, achieving 92.46% mean accuracy for maize populations at 46.2M

parameters. These studies demonstrate that while Transformer architectures deliver

breakthrough performance, they often suffer from parameter explosion, severely limiting

deployment in resource-constrained scenarios.

To address the contradiction between parameter quantity and segmentation accuracy

in plant point cloud segmentation, this study proposes KAN-GLNet, a

Kolmogorov-Arnold segmentation network equipped with global-local feature

modulation. The model is designed to achieve high-precision segmentation under

low-parameter constraints. The key innovations include:

(i)Constructing the first NeRF-derived rapeseed silique point cloud dataset (50

samples), expanding sample scale through data augmentation strategies.

(ii)Proposing reverse bottleneck KAN convolution (enhanced Kolmogorov-Arnold

convolution) to replace conventional convolutions for strengthening feature extraction

capability.

(iii)Designing GLFN multi-scale feature modulation blocks to optimize spatial

representation of complex silique structures by fusing global-local attention mechanisms.

(iv)Introducing the ContraNorm contrastive normalization module to enhance point

cloud feature stability and segmentation consistency.

(v)Proposing an optimized DBSCAN workflow algorithm to achieve automated

silique instance counting.

**-----------------------------------------------------------------------------------------------------------------**

Fig 8

**Comment 2:** *Notably, there is no mention of a validation set being used during training, which would be important for monitoring model generalization and preventing overfitting.
Potential Data Leakage: The test set is selected from the same pool of 220 samples, of which 170 were generated through data augmentation. Since only 50 original images exist, there is a high likelihood that augmented versions of the same original images may have been used across both the training and test sets. This raises concerns about potential data leakage, which could artificially inflate the reported performance. Although the proposed model may offer value, the experimental setup makes it difficult to assess its true effectiveness. Addressing the issues of overfitting and potential data leakage will be critical to strengthen the validity of the results.*

**Authors Response:** We sincerely appreciate your attention to this issue. For the canola point cloud data processing, we adopted a data partitioning strategy following the classic S3DIS dataset approach. Specifically, the S3DIS dataset comprises 271 indoor scene point clouds from six areas (Area_1 to Area_6), with its original partitioning scheme dividing the data into training and test sets: during the validation phase, downsampled sub-point clouds from the test set were used as the validation set, with final evaluation conducted on the complete test set. We implemented a similar data processing pipeline.

As you pointed out, this approach has certain limitations, especially the lack of a dedicated validation set, which makes it difficult to accurately monitor model generalization and overfitting risks. To address this, we improved the data partitioning scheme by strictly dividing the canola point cloud data into training, validation, and test sets at a ratio of 7:2:1. Furthermore, to prevent data leakage, we performed 10-fold data augmentation separately within each subset after partitioning, ensuring that augmented samples do not appear across different subsets and thus enhancing the validity of the experimental results.

We believe this revised partitioning scheme effectively addresses concerns about overfitting and potential data leakage, enabling a more accurate and reliable assessment of model performance. The detailed data partitioning and augmentation process can be found in the “Experiments” section, pages 324–343 of the revised manuscript.

**-----------------------------------------------------------------------------------------------------------------**

The difficulty of acquiring 3D point cloud data is significantly higher than that of 2D

images, which is mainly reflected in two aspects: first, it requires specialized sensors and

specific acquisition methods, and second, the complex structure of plants necessitates

extensive manual annotation efforts ^[42]^. Data augmentation techniques can effectively

alleviate the issue of insufficient training data, providing ample data support for model

optimization. The PointNext study confirmed the effectiveness of basic augmentation

strategies such as rotation and translation in improving model performance. Yao et

al. ^[43]^ employed random point deletion, noise addition, and scaling for point cloud data

of tomato plants across three key growth stages (seedling, flowering, and fruiting). Xie

et al. ^[44]^ successfully expanded the dataset size to 15 times its original scale by

applying operations such as point cloud cropping and jittering to public datasets like

Plant3D ^[45]^.

Based on previous research experience and computational efficiency considerations,

this study adopts a rigorous data processing workflow: first, the NeRF canola dataset is

divided into training, validation, and test sets at a ratio of 7:1:2, followed by 10-fold

data augmentation for each subset. Splitting the dataset before performing

augmentation is to mitigate potential data leakage risks. The specific augmentation

strategy includes four dimensions: randomly discarding 0–30% of point cloud data,

random rotation along the Z-axis between -180° and 180°, random scaling at a ratio of

0.9–1.1, and a 20% probability of flipping along the Y-axis.

**-----------------------------------------------------------------------------------------------------------------**

**Once again, we sincerely thank you for your thoughtful comments and constructive suggestions, which have been invaluable in improving the quality and clarity of our manuscript.**
