# Supplementary material for: KAN-GLNet: An enhanced PointNet++ model for canola silique segmentation and counting
Source: PLoS One. 2025 Nov 17;20(11):e0336622. doi: 10.1371/journal.pone.0336622 (PMC12622843; doi:10.1371/journal.pone.0336622)
Supplement: Attachment — Submitted filename: Response_to_Reviewers_auresp_2.docx [file pone.0336622.s002.docx]

**PLOS ONE**

**Manuscript ID：PONE-D-25-17533R1**

KAN-GLNet: An Enhanced PointNet++ Model for Canola Silique Segmentation and Counting

Jiajun Liu1 , Bei Zhou^1,2*^, Jie Liu^3^ , Xike Zhang^1^ , Jiangshu Wei^1,2^, Yao Zhang^1^ , Junjie

Wu^1^ , Changping Wu^3^ , Di Hu^1^

1 College of Information Engineering, Sichuan Agricultural University, Yaan 625000, China

2 Sichuan Key Laboratory of Agricultural Information Engineering, Sichuan Agricultural University, Yaan 625000, China

3 College of Agronomy, Sichuan Agricultural University, Chengdu 610000, China

* Corresponding author: [12801@sicau.edu.cn](mailto:12801@sicau.edu.cn)

In the revised manuscript, we will use the following formatting: reviewer comments in black italics, our responses in black regular font, and revisions in the manuscript in blue regular font, ensuring clear distinction.

**Response to Reviewer 3:**

**Comment 1:** *In the revised manuscript, the authors increased the number of original samples from 220 (augmented from 50) to 550. However, this dataset size is still far too small for training a model with 5 million parameters.*

**Authors Response:** Dear Reviewer, thank you very much for your attention to our work. We fully understand your concern regarding the balance between the number of model parameters (5.72M) and the dataset size (50 original point cloud samples, augmented to 500 point cloud samples). In the field of deep learning, sufficient training data is indeed one of the key factors to ensure the generalization ability of a model. However, we would like to further explain from the following perspectives that, within the context of this study, the dataset size we adopted is reasonable and effective.

1. Point cloud data has high information density, and the validity of samples differs from that of 2D images.Unlike 2D images, each canola point cloud sample in our dataset was generated through multi-view scanning and 3D reconstruction, with each sample containing 200,000–300,000 3D points, providing rich geometric and structural information. Therefore, point cloud tasks generally place more emphasis on data quality and completeness of information, rather than merely pursuing a large number of samples. This characteristic has been demonstrated by widely used point cloud datasets, for example:

①S3DIS, which contains only 6 large-scale indoor scene point clouds, yet has become one of the benchmark datasets in semantic segmentation;

②Semantic3D, which consists of only 15 outdoor scene point clouds, but has supported numerous high-precision segmentation studies.
Our dataset includes 50 high-quality original point clouds, each with abundant phenotypic structures and detailed information, aligning with the requirement in point cloud tasks that quality is more important than quantity.

2.Plant point cloud datasets are generally limited in scale but sufficient to support effective model training.In the field of plant phenotyping research, due to the high cost of data acquisition and the complexity of reconstruction, public datasets are usually small in scale. For example:

Rice3DSeg, which contains 40 rice point clouds and has been successfully applied to stem–leaf segmentation tasks;PLANesT-3D, which includes 34 plant samples and enables multi-organ segmentation;LAST-Straw, which comprises 84 strawberry point clouds and effectively supported phenotypic parameter extraction.
These precedents demonstrate that in plant 3D analysis, limited but high-quality datasets can achieve reliable results. Our 50 original canola point clouds are comparable in scale to the above datasets, and their diversity has been further enhanced through augmentation, in line with common practices in the field.

3.The model parameter scale is reasonably designed and consistent with current trends in segmentation architectures.Current point cloud segmentation models commonly face a trade-off between parameter size and performance. For example:

①PointNet++ (about 1M parameters), although lightweight, has limited segmentation performance;

②Point Transformer V1/V2/V3 and other self-attention-based models achieve strong performance but involve larger parameter sizes (12M–40M).
Our model, with 5.72M parameters, falls within a medium scale, which avoids the lack of expressive power in lightweight models while also mitigating the risk of over-parameterization. During training, the loss function converged stably and validation performance was satisfactory, indicating that the current data volume is sufficient for the model to learn generalized feature representations.

In conclusion, considering the high information density of point cloud data, the established practices in plant phenotyping datasets, and the actual performance of our model, we believe that our dataset size (50 original point cloud samples, augmented to 500 point cloud samples) is adequate to effectively support the conclusions of our study with a 5.72M-parameter model.

**-----------------------------------------------------------------------------------------------------------------**

**Comment 2:** *Since the authors rely solely on static data augmentation, the diversity introduced is inherently limited, and moreover, data augmentation is typically not applied to validation and test sets.*

**Authors Response:** Dear Reviewer, thank you for your valuable comments on the data augmentation methods in our work. The two issues you raised, namely “the diversity introduced by static data augmentation is limited” and “data augmentation is typically not applied to validation and test sets”, are of great academic value, and we fully acknowledge the importance of these concerns. We now provide a systematic explanation and clarification regarding the points you mentioned.

Regarding the first issue, we completely understand your concern about the limited diversity of static data augmentation methods. It is important to note that in the field of point cloud processing, especially in agricultural scenarios, the augmentation strategies we adopted (including random dropping, rotation, scaling, and flipping) have been extensively validated as effective and standard practices in many top-tier studies. For example, the paper “PointNext: Revisiting PointNet++ with Improved Training and Scaling Strategies” published at NeurIPS 2022 systematically demonstrated that such simple and general augmentation methods, when combined with modern network architectures, can achieve state-of-the-art performance on standard benchmarks such as ModelNet40 and S3DIS. The core idea is that these transformations effectively simulate physical uncertainties in real point cloud data (e.g., sensor noise, object pose variations, and scale differences), thereby helping models learn rotation-invariant, scale-invariant, and occlusion-robust features.

Our canola point cloud dataset also benefits from this strategy. These augmentation operations accurately simulate the actual conditions that may occur during field data collection (such as variations in plant posture, scanning viewpoints, and occlusions), significantly enhancing the model’s generalization ability in real-world scenarios, rather than simply expanding the sample size. Furthermore, the review article “Comprehensive review on 3D point cloud segmentation in plants” published in Artificial Intelligence in Agriculture in 2025 also clearly pointed out that methods such as rotation, flipping, scaling, and down-sampling remain the mainstream techniques widely used in plant point cloud data augmentation.

Regarding the second issue, i.e., the application of data augmentation to validation/test sets, we are especially grateful for your question, which prompted us to further reflect on and clarify our experimental design. We fully agree with the fundamental principle of machine learning that model generalization ability should be evaluated on original, non-augmented data. However, the objective of this study is to assess model robustness, rather than merely standard generalization performance, which is why we adopted a special evaluation process.

The specific design is as follows: during the training phase, we augmented the training set tenfold to help the model learn richer feature representations. During the evaluation phase, we also applied tenfold augmentation to the validation and test sets, with the aim of systematically assessing the model’s stability and output consistency under input perturbations. The final reported performance metrics (e.g., average mIoU) represent the average predictions of the model across each original test sample and its nine augmented versions.

In addition, due to the small size of our dataset (only 50 samples), the original test set contained only 5 samples, making the evaluation results highly susceptible to the randomness of a single split, with high variance. By generating multiple augmented versions for each test sample, we constructed a larger and statistically more stable test set, thereby obtaining more reliable and representative performance estimates and identifying highly robust models that are insensitive to noise and transformations.

We explicitly acknowledge that this method differs from the standard process of purely evaluating generalization ability. The core objective of this study is to answer the robustness question: “Does the model perform consistently when faced with the inevitable data variations in the real world?” We have added clarifications on this design in the limitations section of the paper.

In summary, we believe that the augmentation methods we employed are well justified in both theory and practice, and that the special experimental design for robustness evaluation effectively supports the achievement of this study’s objectives. Once again, thank you for your valuable comments, which have provided important guidance for us to further improve our research work.

**-----------------------------------------------------------------------------------------------------------------**

This study also has certain limitations in the application of data augmentation strategies. Although the static augmentation methods employed (such as rotation, scaling, and random dropout) have been widely validated in the field of point cloud processing and can effectively simulate real-world disturbances such as pose variations and occlusions during field data acquisition, the diversity of the generated data remains constrained by the predefined transformation space, making it difficult to cover the complex variations present in all real scenarios. Moreover, to evaluate the robustness of the model when facing input perturbations, this study also applied data augmentation to the validation and test sets. While this design improved the stability of statistical evaluation under small-sample conditions and allowed a sharper focus on answering the robustness question of “whether the model performs consistently under real disturbances,” the results may not be directly comparable to traditional generalization performance evaluations based solely on original data. Future work could further introduce dynamic augmentation techniques and also provide evaluation results based on the original test set, so as to more comprehensively reflect the performance of the model.

**-----------------------------------------------------------------------------------------------------------------**

**Comment 3:** *In the previous review round, the authors were specifically asked about techniques to prevent overfitting in such scenarios, yet they did not provide a clear, substantive answer—merely citing a few other studies without demonstrating their own implementation. Some of these cited studies also applied deep learning to very small sample sizes in ways that are arguably questionable, suggesting that these works may not have been rigorously assessed by their reviewers and editors. For small-sample training, a more robust strategy would be to first pre-train the model on a large labeled dataset, then fine-tune it on the small dataset with dynamic data augmentation to increase diversity and reduce overfitting.*

**Authors Response:** Dear Reviewer, thank you very much for your valuable comments once again, and we sincerely apologize for any unclear expressions in our previous response. We fully understand your deep concern regarding overfitting in small-sample learning. Here, we aim to provide a clearer and more substantive explanation of the specific technical strategies used in our work, to demonstrate that rigorous and effective measures have been taken to mitigate overfitting.

Our anti-overfitting strategy is a systematic approach that spans three levels: data, training strategy, and model regularization:

Data level: targeted augmentation and preprocessing.

The static data augmentations we employed (random dropout, rotation, scaling, and flipping) are designed for purposes far beyond merely expanding the sample size. Each operation is carefully crafted to accurately simulate the physical uncertainties present in field data acquisition, such as sensor noise, plant pose variations, and scale differences. This forces the model to learn intrinsic features that are invariant to such perturbations, rather than memorizing specific noise in the training samples, thereby enhancing generalization from the data source itself.

Training strategy and hyperparameter optimization: explicit regularization design.

In our training configuration, we adopted multiple widely validated regularization techniques:

Optimizer and Weight Decay: We chose the AdamW optimizer with a relatively high weight decay coefficient (0.01). The key distinction between AdamW and traditional Adam is that AdamW decouples weight decay from gradient updates, effectively constraining the magnitude of model weights, significantly mitigating overfitting and promoting convergence to flatter minima.

Adaptive learning rate scheduling: We employed a Cosine Annealing learning rate schedule. This strategy smoothly decays the learning rate from the initial value (0.0001) to the minimum value (0.00001). Such dynamic decay helps the model converge more stably to an optimal solution in the later stages of training, avoiding oscillations near the optimum and potential overfitting caused by a fixed learning rate.

Model evaluation and result validation.

The most direct evidence is that the training loss and validation loss curves of our model throughout 250 training epochs exhibit highly consistent and synchronized downward trends, converging without any noticeable divergence (i.e., no increase in validation loss). This phenomenon provides the strongest empirical proof that the model does not experience severe overfitting, indicating that the learned features possess good generalization ability.

Finally, we fully agree with your suggestion that a “pretraining–fine-tuning” paradigm is an ideal solution for small-sample learning. However, in the specific domain of plant point cloud processing, particularly for crops such as canola, there is currently a complete lack of publicly available large-scale, finely annotated point cloud datasets suitable for pretraining. This objective limitation has led mainstream studies in the field (e.g., tomato, maize, cotton point cloud analysis) to adopt “train from scratch” strategies combined with strong regularization techniques. Our work, under this realistic constraint, seeks to obtain a robust and reliable model through the comprehensive technical measures outlined above.

We sincerely appreciate your review. Your comments have greatly improved both the rigor and clarity of our work.

**-----------------------------------------------------------------------------------------------------------------**

**Comment 4:** *Then, comparing the first and second submissions, the first version contained a data leakage issue. While this was addressed in the revised version, the model’s performance somehow improved, which raises serious doubts about the validity of the reported results.*

**Authors Response:** Dear Reviewer, thank you very much for your continued attention and rigorous review. The questions you raised are crucial to ensuring the scientific validity of our research results. We fully understand your concerns regarding performance changes and hereby provide a clear explanation.

First, we solemnly state that in the revised version, we have thoroughly resolved the potential data leakage issue present in the initial submission by implementing a strict re-division of the dataset. The training set, validation set, and test set are completely independent, and all augmentation operations were performed only on the training set. This forms the fundamental premise of the validity of our results.

Regarding the phenomenon that performance improved after eliminating the leakage issue, this is not an anomaly but rather a reasonable outcome resulting from multiple positive improvements acting together:

1. **Optimization of training hyperparameters:** Compared with the initial version, we carried out a series of key hyperparameter adjustments to improve model performance, mainly including:

* Increasing the **Batch Size from 8 to 16**. A larger batch size helps provide more stable gradient estimates, making the optimization process smoother, which facilitates the model escaping local minima and converging to solutions with stronger generalization ability.

* Employing a **Cosine Annealing learning rate schedule**. This strategy dynamically adjusts the learning rate and enables finer tuning toward the end of training, thereby achieving better model performance.

* Using the **AdamW optimizer with Weight Decay set to 0.01**. These settings explicitly introduce regularization constraints, effectively alleviating overfitting and thus enhancing the model’s generalization performance on the test set.

2. **Effective expansion of training data:** Under the condition of strictly preventing leakage, we expanded the augmented training set from 220 samples to 500 samples. While the validation and test sets remained independent, the more diverse training data enabled the model to learn more robust feature representations, which is another reasonable reason for the observed performance improvement.
3. **Experimental rigor and reproducibility:** We assure you that all our experiments and ablation studies (for fairness, baseline models used the hyperparameters provided in their original papers) were conducted under exactly the same hyperparameter settings and training environment, ensuring comparability and fairness of results. Most importantly, to further substantiate the authenticity and rigor of our conclusions, we have supplemented our open-source repository with the complete training weights and detailed training/testing logs of all comparison models and ablation experiments from this revision. We kindly invite you to verify and supervise. All results can be strictly reproduced.

In summary, the performance improvement did not result from data leakage but rather from our positive optimizations in training strategies and the use of more adequate training data. Once again, we apologize for the confusion caused by the improper data split in the initial version, and we sincerely thank you for your critical questions, which have made our work more rigorous and reliable.

**-----------------------------------------------------------------------------------------------------------------**

**Comment 5:** *Additionally, there are inconsistencies between the text and the figures/tables. For example, the manuscript mentions using an RTX 4090 in the main text, whereas the corresponding table lists an RTX 3090. Such discrepancies undermine the clarity and reliability of the reported experimental setup.*

**Authors Response:** Dear Reviewer, thank you very much for reviewing our manuscript with such rigor and for pointing out this critical inconsistency. We sincerely apologize for this oversight, which indeed affected the clarity of the manuscript, and we take full responsibility for it.

You are absolutely correct about the issue you raised. Here we provide a complete explanation:

Our initial experiments were indeed conducted on a single NVIDIA GeForce RTX 4090 (24GB) GPU. However, during the first revision, based on the valuable comments from you and another reviewer, we re-divided the dataset and carried out a comprehensive and thorough set of new experiments, including all main experiments, comparative experiments, and extensive ablation studies.

To efficiently complete this large volume of computation in a short time, we rented multiple cloud servers. We prioritized ensuring consistency across all experimental environments:

Hardware consistency: All rented servers were equipped with NVIDIA GeForce RTX 3090 (24GB) GPUs. Their memory capacity is identical to that of the previously used RTX 4090, ensuring that key training settings such as maximum batch size did not need to be changed.

Software environment consistency: The operating system, CUDA version, PyTorch version, Python version, and all dependencies were kept absolutely identical across all servers, created from a single standardized image. This guaranteed a high level of reproducibility for the experimental results.

We chose the RTX 3090 for project budget considerations, but its computational power and memory capacity were fully sufficient for all the requirements of this study. Moreover, all experiments were completed under identical hardware and software configurations, ensuring fairness and comparability of all results.

Unfortunately, during the final preparation of the manuscript, we overlooked this point and failed to update all hardware descriptions in the text consistently to the actual RTX 3090 used, which caused the contradiction between the text and the tables. This was entirely our proofreading error.

We have now unified all relevant descriptions in the manuscript to “NVIDIA RTX 3090.” Once again, we sincerely thank you for your outstanding insight and rigorous review. Your comments have greatly improved the accuracy and reliability of our work.
